# The Anti-Atopic Dermatitis Effects of *Mentha arvensis* Essential Oil Are Involved in the Inhibition of the NLRP3 Inflammasome in DNCB-Challenged Atopic Dermatitis BALB/c Mice

**DOI:** 10.3390/ijms24097720

**Published:** 2023-04-23

**Authors:** So-Yeon Kim, Arjun Sapkota, Young Joo Bae, Seung-Hyuk Choi, Ho Jung Bae, Hyun-Jeong Kim, Ye Eun Cho, Yu-Yeong Choi, Ju-Yeon An, So-Young Cho, Sun Hee Hong, Ji Woong Choi, Se Jin Park

**Affiliations:** 1 Department of Food Biotechnology and Environmental Science, Kangwon National University, Chuncheon 24341, Republic of Korea; 2 College of Pharmacy and Gachon Institute of Pharmaceutical Sciences, Gachon University, Incheon 21936, Republic of Korea; 3 Agriculture and Life Science Research Institute, Kangwon National University, Chuncheon 24341, Republic of Korea; 4 School of Applied Science in Natural Resources & Environment, Hankyong National University, Anseong 17579, Republic of Korea; 5 School of Natural Resources and Environmental Sciences, Kangwon National University, Chuncheon 24341, Republic of Korea

**Keywords:** *Mentha arvensis*, atopic dermatitis, NLRP3 inflammasome, BMDMs, IL-1β

## Abstract

The NLRP3 inflammasome is upregulated by various agents, such as nuclear factor-kappa B (NF-κB), lipopolysaccharide (LPS), and adenosine triphosphate (ATP). The NLRP3 inflammasome facilitations the maturation of interleukin (IL)-1β, a proinflammatory cytokine that is critically involved in the pathogenesis of atopic dermatitis (AD). Although the NLRP3 inflammasome clearly exacerbates AD symptoms such as erythema and pruritus, drugs for AD patients targeting the NLRP3 inflammasome are still lacking. Based on the previous findings that *Mentha arvensis* essential oil (MAEO) possesses strong anti-inflammatory and anti-AD properties through its inhibition of the ERK/NF-κB signaling pathway, we postulated that MAEO might be capable of modulating the NLRP3 inflammasome in AD. The aim of this research was to investigate whether MAEO affects the inhibition of NLRP3 inflammasome activation in murine bone marrow-derived macrophages (BMDMs) stimulated with LPS + ATP in vitro and in a murine model displaying AD-like symptoms induced by 2,4-dinitrochlorobenzene (DNCB) in vivo. We found that MAEO inhibited the expression of NLRP3 and caspase-1, leading to the suppression of NLRP3 inflammasome activation and IL-1β production in BMDMs stimulated with LPS + ATP. In addition, MAEO exhibited efficacy in ameliorating AD symptoms in a murine model induced by DNCB, as indicated by the reduction in dermatitis score, ear thickness, transepidermal water loss (TEWL), epidermal thickness, and immunoglobulin E (IgE) levels. Furthermore, MAEO attenuated the recruitment of NLRP3-expressing macrophages and NLRP3 inflammasome activation in murine dorsal skin lesions induced by DNCB. Overall, we provide evidence for the anti-AD effects of MAEO via inhibition of NLRP3 inflammasome activation.

## 1. Introduction

The skin is constantly exposed to pathogens and environmental risks, including pollutants, particulate matter (PM), and heavy metals, and is prone to skin disorders [1]. Atopic dermatitis (AD) is a common skin disorder that is characterized by symptoms such as erythema, redness, swelling, dryness, and persistent itching. The prevalence of AD has increased in recent years due to urbanization and industrialization, with many experiencing continuous relapses until adulthood [2]. AD results from an inappropriate innate immune response attempting to protect the host. The immune system directly senses intracellular danger signals via nuclear factor-kappa B (NF-κB) and nucleotide-binding oligomerization domain-like receptors (NLRs) that recognize pathogen-associated molecular patterns [3,4,5]. NF-κB regulates the inflammatory response and is activated by mitogen-activated protein kinases (MAPKs) [6]. In addition, the NLR is considered a novel variable for the systemic inflammatory response to various infections or stimuli [7]. NLR family pyrin domain containing 3 (NLRP3) protein, a well-known inflammasome sensor, combines with a connector molecule called apoptosis-associated speck-like protein containing a caspase activation and recruitment domain (ASC) [8]. Cytokines and chemokines released by these signaling pathways are important for inflammation mediated by immune cells and skin barrier functions [9].

Upon stimulation with various agents, including lipopolysaccharide (LPS) and adenosine triphosphate (ATP), large cytosolic multiprotein complexes called inflammasomes are formed [10]. The NLRP3 inflammasome is a crucial inflammatory component of the innate immune system responsible for activating caspase-1 and producing the proinflammatory cytokine interleukin (IL)-1β and IL-18 [11]. In addition, activated NLRP3 inflammasome results in a downstream response in cell death called pyroptosis [12]. IL-1β belongs to the IL-1 family of cytokines and is a significant contributor to the development of chronic inflammatory skin disorders, such as AD [13]. In addition, IL-1β can reprime the NLRP3 inflammasome, which may exacerbate chronic inflammatory conditions [14]. Another member of the IL-1 family, IL-18, is a protein that regulates inflammatory responses in the immune system and plays a vital role in the NLRP3 inflammasome-mediated inflammatory response [15]. IL-18 is implicated in contact dermatitis, psoriasis, and atopic dermatitis because it regulates T helper (Th)1 and Th2 cells to overexpress cytokines [16]. In fact, the NLRP3 inflammasome has been associated with the development of metabolic disorders, including gout, obesity, type 2 diabetes, and inflammatory diseases such as arthritis [17]. Furthermore, NLRP3 inflammasomes have been associated with various inflammatory and autoimmune skin disorders, including dermatitis, psoriasis, and acne [18]. In particular, expression of the NLRP3 inflammasome can contribute to AD and lead to skin colonization and infection [19]. It has been increasingly elucidated that the NLRP3 inflammasome secretes IL-1β and aggravates AD symptoms [20]. Therefore, targeting the NLRP3 inflammasome may represent a promising therapeutic strategy for AD management [21].

The anti-inflammatory properties of *Mentha arvensis* essential oil (MAEO) were demonstrated in our previous study, in which it was found to be effective against LPS-induced RAW 264.7 macrophages and HaCaT keratinocytes. Moreover, we demonstrated that MAEO inhibited the ERK/NF-κB signaling pathways and significantly improved AD-like lesions. Notably, NF-κB is a critical transcription factor that is involved in upregulation of the NLRP3 inflammasome [22]. Despite the demonstrated anti-atopic properties of MAEO, the underlying mechanisms involving the NLRP3 inflammasome activation are still unclear. Our study aimed to investigate whether MAEO could affect NLRP3 inflammasome activation using murine bone marrow-derived macrophages (BMDM) stimulated with LPS + ATP and an AD animal model induced by 2,4-dinitrochlorobenzene (DNCB).

## 2. Results

### 2.1. Chemical Analysis of MAEO

The GC-MS analysis of MAEO allowed us to identify a total of 45 compounds based on their retention times and mass spectral data, as shown in Table 1. Similar to that described by Kim et al. [23], menthol (35.67%), menthone (26.19%), and piperitone (13.73%) are major components in MAEO. These three compounds have reported anti-inflammatory, antibacterial, and antitumor effects and are found in most species of the *Mentha* genus [24,25,26].

### 2.2. The Effects of MAEO on NLRP3 Inflammasome Formation in BMDMs

**Figure 1:**
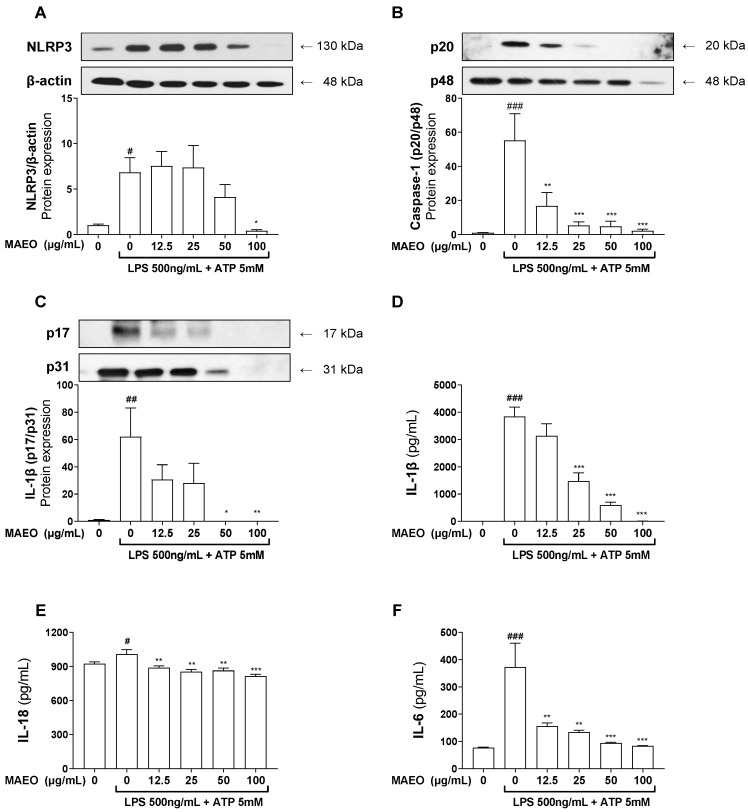
Effects of MAEO on the formation and activation of the NLRP3 inflammasome in BMDMs. MAEO was pretreated 30 min before the induction of inflammation, and LPS (500 ng/mL) was used to stimulate inflammation for 4 h followed by ATP (50 mM) addition. Relative protein expression of (**A**) NLRP3, (**B**) caspase-1, and (**C**) IL-1β was quantified. The levels of (**D**) IL-1β, (**E**) IL-18, and (**F**) IL-6 were determined in culture medium. One-way ANOVA was used for statistical analysis, and significance was established as # *p* < 0.05, ## *p* < 0.01, ### *p* < 0.001 versus the control group; * *p* < 0.05, ** *p* < 0.01, *** *p* < 0.001 versus the LPS + ATP-stimulated group.

We investigated the effects of MAEO on the expression of NLRP3 and the formation of inflammasomes in LPS-primed BMDMs by pretreating cells with different concentrations of MAEO (12.5–100 μg/mL) before stimulation with LPS (500 ng/mL, 4 h) and exposure to ATP (50 mM) for an additional 30 min. LPS + ATP-primed cells showed increased NLRP3 expression, whereas 100 μg/mL MAEO pretreatment significantly suppressed NLRP3 expression (Figure 1A). NLRP3 inflammasome formation converts pro-caspase-1 into its active form, leading to the cleavage of pro-IL-1β into mature active IL-1β [27]. Therefore, we investigated the expression of caspase-1 and IL-1β to confirm that MAEO can regulate NLRP3 inflammasome formation. As expected, pretreatment with MAEO dose-dependently reduced the LPS + ATP-induced increase in active caspase-1 (Figure 1B). Similarly, LPS + ATP-stimulated cells expressed active IL-1β, which disappeared in both the pro- and active forms above 50 μg/mL MAEO (Figure 1C). We noted that MAEO considerably decreased the production of the proinflammatory cytokines IL-1β and IL-18, which are induced by NLRP3 inflammasomes (Figure 1D,E). Additionally, similar to our prior research, MAEO also reduced the level of IL-6 in the culture medium (Figure 1F). These findings suggest that MAEO may have anti-inflammatory properties by controlling NLRP3 expression and regulating inflammasome formation.

### 2.3. The Effects of MAEO on DNCB-Induced AD-like Clinical Symptoms in BALB/c Mice

**Figure 2:**
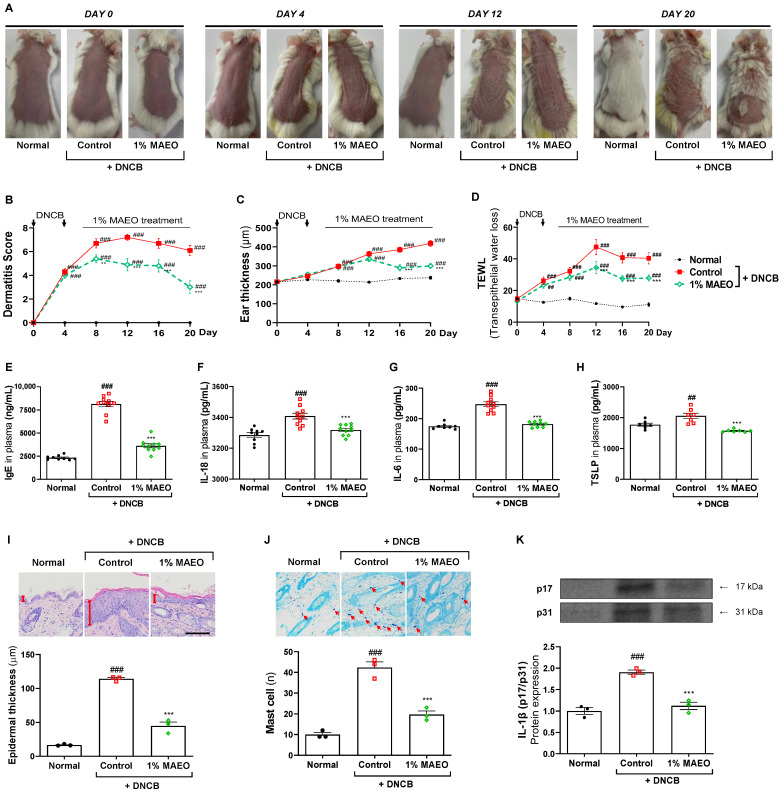
Effects of MAEO on AD-like skin lesions induced by DNCB in BALB/c mice. (**A**) Representative images of the mice were captured at the same magnification. (**B**) The severity of dermatitis, (**C**) thickness of the ear, and (**D**) TEWL were measured every four days. Two-way ANOVA was used for statistical analysis, and significance was established as ### *p* < 0.001 versus the control group; *** *p* < 0.001 versus the control group. (**E**) IgE, (**F**) IL-18, (**G**) IL-6, and (**H**) TSLP levels were determined in plasma. Images of the dorsal skin stained with H&E and TB were captured at 200× magnification, and the scale bar is 100 μm. (**I**) Epidermal thickness (red line) and (**J**) the number of infiltrated mast cells (red arrows) were quantified. (**K**) The relative protein expression of IL-1β was quantified. One-way ANOVA was used for statistical analysis, and significance was established as ## *p* < 0.01, ### *p* < 0.001 versus the normal group; *** *p* < 0.001 versus the control group.

Based on the in vitro studies, we investigated the effects of MAEO on the expression of NLRP3 in AD skin lesions using a DNCB-induced AD-like murine model. The SCORAD index and ear thickness were used to evaluate the DNCB-induced AD-like clinical symptoms at 4-day intervals. Treatment with 1% MAEO topically resulted in significant recovery of AD symptoms, which was evident from Day 12 and continued until the final day, as shown in Figure 2A. The SCORAD index, which quantifies clinical symptoms, showed a significant increase in the control group that was treated with the vehicle in contrast to the normal group. As expected, MAEO effectively decreased the dermatitis score, ear edema, and TEWL (Figure 2B–D). We also observed that MAEO decreased the level of IgE in plasma, an important biomarker in determining AD (Figure 2E). In addition, the levels of IL-18 and IL-6, which play central roles in inflammation, were significantly decreased by 1% MAEO (Figure 2F,G). Furthermore, we observed that MAEO was successful in decreasing the level of TSLP, a proinflammatory cytokine that leads to AD (Figure 2H).

Furthermore, we performed histological examinations, including H&E and TB staining, to assess the effects of MAEO on epidermal thickness and mast cell infiltration in the dorsal skin tissue of mice with AD-like symptoms induced by DNCB. The control group had considerably higher levels of epidermal thickness and infiltrated mast cells than the normal group. However, MAEO significantly decreased the DNCB-induced increase in epidermal thickness and infiltrated mast cells (Figure 2I,J). Notably, MAEO effectively decreased the expression of IL-1β infiltration in the dorsal skin tissue of mice with DNCB-induced AD-like symptoms (Figure 2K). These results support and corroborate previous research indicating a significant amelioration of DNCB-induced atopic skin inflammation with 1% MAEO treatment, and a potential link to the modulation of NLRP3 inflammasome activity.

### 2.4. The Effects of MAEO on NLRP3 Inflammasome Formation in Macrophages of DNCB-Induced AD-like Murine Dorsal Skin

**Figure 3:**
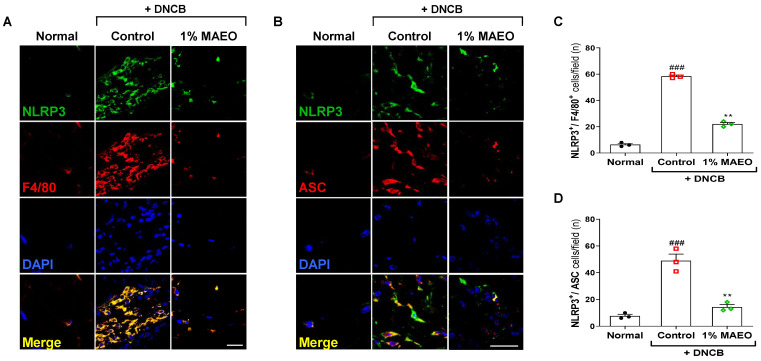
Effects of MAEO on NLRP3 expression and inflammasome formation in a DNCB-induced AD murine model. (**A**) NLRP3^+^/F4/80^+^ and (**B**) NLRP3^+^/ASC^+^ double positive macrophages in the dorsal skin were observed at 400× magnification, and representative images are displayed with a scale bar of 20 μm. For quantification, (**C**) NLRP3^+^/F4/80^+^ cells and (**D**) NLRP3^+^/ASC^+^ cells were manually counted and reported as the total number of cells/unit area (mm^2^) of dermis. One-way ANOVA was used for statistical analysis, and significance was established as ### *p* < 0.001 versus the normal group; ** *p* < 0.01 versus the control group.

Macrophages, the main cell type mediating the NLRP3 inflammasome response, can massively migrate to skin with AD lesions. Therefore, we identified that MAEO has the potential to obstruct inflammasome formation and NLRP3 expression in macrophages found in AD lesions (Figure 3A,B). The DNCB-induced AD model demonstrated a notable increase in the count of cells positive for NLRP3 and F4/80, whereas administration of 1% MAEO significantly attenuated NLRP3-expressing macrophages (Figure 3C). In addition, we observed an increase in double-stained cells positive for NLRP3 and ASC in the dorsal skin of DNCB-induced AD-like mice. As expected, the group treated with 1% MAEO exhibited a statistically significant reduction in the number of cells positive for both NLRP3 and ASC (Figure 3D). These findings indicate that MAEO can effectively regulate both the infiltration of NLRP3-overexpressing macrophages and formation of the NLRP3 inflammasome in DNCB-induced AD lesions.

## 3. Discussion

We demonstrated the anti-inflammatory properties of MAEO, specifically related to the NLRP3 inflammasome, in both LPS + ATP-induced BMDMs and an AD murine model induced by DNCB. AD is a persistent inflammatory condition of the skin that is influenced by several cofactors, including impaired skin barrier function, immune system modifications, and a complex genetic background [28]. The aforementioned factors contribute to the disruption of the epithelial structure, leading to increased epidermal thickness, swelling, redness, and formation of lichenized plaques in AD [29,30]. Inflammatory skin lesions in AD are known to be characterized by the accumulation of macrophages, and many studies on AD have reported that macrophage accumulation is a crucial factor in its pathogenesis [31,32,33]. Excessive and prolonged inflammation activates an inflammatory complex called the NLRP3 inflammasome in macrophages in skin lesions.

The inflammatory response heavily relies on the NLRP3 inflammasome, which includes the adapter molecule ASC, responsible for procaspase-1 recruitment. This inflammasome is primarily activated in proinflammatory macrophages, causing the release of proinflammatory cytokines, such as IL-1β and IL-18 [34]. Therefore, IL-1β and IL-18 might be significant mediators of the AD phenotype through NLRP3 inflammasome activation [35]. IL-1β is a strong stimulator of immune and inflammatory responses that cause immune cell recruitment and activation at sites of inflammation or infection [36]. Numerous studies have reported that IL-1β contributes to skin inflammation in AD and induces IL-6 production both in vitro and in vivo [37,38,39,40]. The improper regulation of IL-6 triggers a cytokine storm associated with various autoimmune disorders, such as AD and asthma [41]. Additionally, IL-18, which belongs to the IL-1 family together with IL-1β, has the ability to regulate immune responses [42]. In fact, IL-6 and IL-18 have been suggested as potential etiologies of AD, as their blood levels have been shown to increase with AD severity [43,44,45,46]. Therefore, regulating the NLRP3 inflammasome and its associated cytokines, including IL-1β, IL-18, and IL-6, is important in preventing inflammatory skin diseases. Moreover, some previous studies have suggested that the NLRP3 inflammasome ultimately triggers pyroptosis, leading to various skin diseases, including AD and acne [14,47]. Mitochondrial cleavage inhibitor 1 (mdivi-1), an NLRP3 inflammasome inhibitor, has been proposed as a therapeutic agent for AD due to its ability to inhibit NLRP3 inflammasome activation and pyroptosis in keratinocytes under AD-like inflammation [48,49]. mdivi-1 was reported to decrease expression of NLRP3 and ASC, cleavage of caspase-1, and mature IL-1β and IL-18 in keratinocytes under AD-like inflammation [49]. Therefore, targeting the NLRP3 inflammasome through inhibitors such as mdivi-1 may be a promising therapeutic approach for AD. Notably, BMDMs can induce inflammation (M1-polarized macrophages) through the release of proinflammatory cytokines in response to various stimuli, such as LPS and endogenous stress. Although the role of LPS in atopic dermatitis is still unknown, there is evidence that LPS-activated NF-κB can determine the severity of AD [50,51]. Additionally, excessive Th1 due to Th1/Th2 imbalance can activate M1-polarized macrophages and worsen AD [52]. Furthermore, ATP acts on P2X7 receptors, a subtype of purine receptor, to induce K^+^ efflux and affect NLRP3 inflammasome formation [53]. In particular, BMDMs are utilized as a suitable model because K^+^ efflux is a general requirement to activate the NLRP3 inflammasome [54,55,56,57]. The findings of this investigation demonstrated that MAEO inhibited the upregulation of NLRP3 and cleavage of caspase caused by LPS + ATP. In addition, MAEO lowered mature IL-1β and production of the proinflammatory cytokines IL-1β and IL-18. Furthermore, MAEO reduced the production of IL-6, which is essential in innate immunity together with the NLRP3 inflammasome. Based on our observations, we hypothesized that MAEO may have the potential to modulate AD by inhibiting development of the NLRP3 inflammasome in BMDMs.

**Figure 4:**
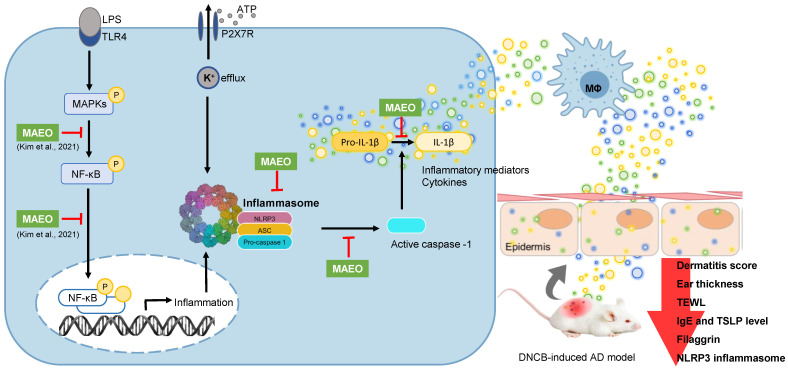
Proposed mechanism for the action of MAEO on the NLRP3 inflammasome under inflammatory conditions induced by LPS + ATP and AD-like conditions induced by DNCB. The mechanism of action of MAEO for MAPK/NF-κB inhibition was reported in our previous study [23].

Expanding on the previous findings, we investigated the potential therapeutic effects of MAEO against AD by assessing its impact on macrophage infiltration and NLRP3 inflammasome activation in vivo. The topical application of MAEO was administered to DNCB-induced AD-like lesions in BALB/c mice, which were repeatedly treated with DNCB on their dorsal skin and ear. As expected, excellent relief effects of 1% MAEO were visually observed and quantified as an index. The DNCB-induced increases in dermatitis score, ear thickness, and TEWL were significantly decreased by 1% MAEO compared to the control group. In addition, 1% MAEO reduced the levels of IgE, IL-18, IL-6, and TSLP in plasma. The main immunological changes in AD are an increase in both IgE and hypersecretion of cytokines [58]. Concomitantly, the level of TSLP in the blood may also increase due to an allergic reaction [59]. Many studies have reported significantly increased levels of IgE, IL-18, IL-6, and TSLP in the plasma of patients or animals with AD [44,45]. In addition, topical MAEO administration had a positive effect on epidermal thickness reduction and mast cell infiltration in the dorsal skin. Additionally, we confirmed that 1% MAEO decreased IL-1β expression in dorsal skin-induced AD. Thus, MAEO was expected to reduce NLRP3 inflammasome activation in AD-like dorsal lesion skin. Furthermore, we found increased accumulation of NLRP3-expressing macrophages and formation of the NLRP3 inflammasome in DNCB-induced AD-like dorsal skin lesions. In particular, confirmation of co-localization of macrophages (F4/80) with NLRP3 and NLRP3 with ASC suggests a link between activation of the NLRP3 inflammasome in macrophages and the severity of AD. MAEO reduced the accumulation of NLRP3-expressing macrophages and development of the NLRP3 inflammasome in dorsal skin lesions. Moreover, 1% MAEO significantly downregulated the expression of the NLRP3 inflammasome in comparison to the control group. In addition to our in vitro results in BMDMs, in vivo results allow us to understand better the mechanisms by which NLRP3 inflammasome expression in macrophages affects AD severity. Thus, MAEO has the potential to suppress both inflammation and NLRP3 inflammasome activation, ultimately leading to its anti-AD effects (Figure 4).

In conclusion, we investigated the effects of MAEO on the activities of the NLRP3 inflammasome in LPS + ATP-induced inflammatory stimulation and DNCB-induced AD-like murine models. As we reported, MAEO, containing menthol and menthone as its primary constituents, possesses potent anti-inflammatory properties and may be a promising candidate for treating AD. Additionally, MAEO could be formulated as a cream or lotion and applied to affected areas to reduce inflammation and alleviate symptoms. Moreover, we found that MAEO effectively ameliorated AD-like symptoms by modulating expression of the NLRP3 inflammasome. Although clinical trials on NLRP3 inflammasome inhibition by MAEO are still lacking, we identified the anti-inflammatory molecular mechanism of MAEO and its ameliorating effect on atopic symptoms through two studies. Modulating the expression of NLRP3 inflammasome by MAEO suggests that inhibiting its activity could be a potential therapeutic strategy for treating inflammatory skin disorders such as psoriasis and eczema. In addition, MAEO is generally considered to contain safe substances and has already been used; thus, MAEO has the advantage of being applicable to humans [60,61]. Therefore, MAEO can be considered as an alternative or complementary therapy to current treatments for AD, such as topical corticosteroids and immunosuppressive agents, to enhance their effectiveness and reduce the side effects. Taken together, these results indicate that MAEO holds considerable promise as a therapeutic intervention for remedying inflammatory skin disorders, with a particular focus on atopic dermatitis, through its capacity to suppress inflammasome activity.

## 4. Materials and Methods

### 4.1. Materials

Minimum essential medium-α (MEM-α), fetal bovine serum (FBS), and penicillin–streptomycin (P/S) were purchased from Life Technologies (Carlsbad, CA, USA). Recombinant mouse macrophage colony-stimulating factor (M-CSF) and enzyme-linked immunosorbent assay (ELISA) kits for interleukin-1β (IL-1β) were obtained from R&D Systems (Minneapolis, MN, USA). An ELISA kit for immunoglobulin E (IgE), interleukin-6 (IL-6), interleukin-18 (IL-18), and thymic stromal lymphopoietin (TSLP) was supplied by Invitrogen (Carlsbad, CA, USA). Lipopolysaccharide from *Escherichia coli* O26:B6 (LPS), adenosine 5′-triphosphate disodium salt hydrate (ATP) and 2,4-dinitrochloro benzene (DNCB) were purchased from Sigma Chemical Co. (St. Louis, MO, USA). Radioimmunoprecipitation assay buffer (RIPA buffer) and skim milk powder were supplied by ELPIS Biotech (Deajeon, Republic of Korea) and BD Difco (Sparks, MD, USA), respectively. All other materials and reagents used in the study were of the highest quality available.

### 4.2. Mentha arvensis Essential Oil

As previously described by Kim et al. [23], MAEO was prepared. Our previous research demonstrated that MAEO exhibited anti-inflammatory properties without cytotoxicity at 100 μg/mL in both RAW 264.7 macrophages and HaCaT keratinocytes. In addition, 1% MAEO had anti-atopic effects in the DNCB-induced AD murine model. Therefore, to ensure consistency with previous experiments [23], we used the same concentration for all investigations in this study. For in vitro treatment, MAEO was dissolved in dimethyl sulfoxide to a final concentration of 0–100 μg/mL, resulting in a transparent solution with a unique spicy scent. For in vivo experiments, MAEO was dissolved in olive oil at a concentration of 1% (*v*/*v*) and vortexed, resulting in a sticky, translucent yellow solution with a spicy scent. The solvent used affected the color and viscosity of the resolution, while the distinctive spicy scent was consistent.

### 4.3. Gas Chromatography-Mass Spectrometry

The Kim et al. [23] method was used to determine the various compounds present in MAEO. MAEO was analyzed using a Varian CP3800 gas chromatograph and a Varian 1200 L mass detector (Varian, Inc., Palo Alto, CA, USA) fitted with a polydimethylsiloxane-modified VF-5MS capillary column (30 m × 0.25 mm × 0.25 μm). The sample was heated from 50 °C to 250 °C at a 5 °C/min rate in the oven. The ionization detector and injector temperature were adjusted to 200 °C and 250 °C, respectively. Helium was employed as the carrier gas, with a constant flow rate of 1 mL/min. A split ratio of 10:1 was used to inject 2 μL of the sample, and the mass spectrum was obtained using a 70 eV electron ionization energy system that scanned the range of 50–500 m/z. The compound was identified by comparing its linear retention index (LRI) obtained from gas chromatography with reference spectra from the National Institute of Standards and Technology (NIST, 3.0) and available literature data [62]. Chemical standards, including menthol, menthone, and piperitone were obtained from Sigma Chemical Co. (St. Louis, MO, USA). To quantify the major compounds in MAEO, a standard solution containing the main compound was injected in a volume of 1.0 μL at an appropriate concentration. The major constituents were determined in triplicate, and their concentrations were determined by plotting calibration curves using their peak areas.

### 4.4. Animals

The animal experiments were conducted in accordance with the Institutional Animal Care and Use Committee (IACUC) guidelines of the Laboratory Animal Research Center at Kangwon National University, Korea (KW-200122-2). Six-week-old female BALB/c mice (14–15 g) were procured from the Orient Bio Experimental Animal Breeding Center (Seongnam, Republic of Korea) and housed in cages with 5 animals each under standard conditions, including a 12 h light–dark cycle, a constant humidity of 45–65%, and unrestricted access to food and water. A total of 30 mice were utilized for the study, with 10 mice allocated to each group.

### 4.5. Isolation and Differentiation of Mouse BMDMs

Bone marrow macrophages were isolated, according to Gaire et al. [63]. Briefly, femurs and tibias of 8-week-old ICR mice were flushed to obtain bone marrow cells. Then, the cells were differentiated into adherent bone marrow-derived macrophages (BMDMs) in minimum essential medium-α (MEM-α) with 10% heat-inactivated fetal bovine serum (FBS), 100 units/mL penicillin-streptomycin (P/S), and 30 ng/mL recombinant mouse macrophage colony-stimulating factor (M-CSF) at 37 °C and 5% CO_2_. BMDMs were exposed to different concentrations (12.5–100 μg/mL) of MAEO for 1 h before being stimulated with 500 ng/mL lipopolysaccharide (LPS) for 4 h and given an additional stimulation with 50 mM adenosine triphosphate (ATP) for 30 min.

### 4.6. Enzyme-Linked Immunosorbent Assay (ELISA)

ELISA kits from R&D Systems (Minneapolis, MN, USA) and Invitrogen (Carlsbad, CA, USA) were used to determine the levels of IL-1β, IgE, IL-18, IL-6, and TSLP in the culture medium or plasma, following the manufacturer’s instructions. The collected cell culture medium and plasma were used as samples after centrifugation at 1500 rpm and 10,000 rpm for 5 min, respectively. The optical density at 450 nm was measured using a SpectraMax 190 microplate reader (Molecular Devices, San Jose, CA, USA).

### 4.7. Western Blot Analysis

Total cellular proteins were extracted from bone marrow-derived macrophages (BMDMs) or dorsal skin using RIPA buffer (ELPIS Biotech, Deajeon, Republic of Korea) with phosphatase and protease inhibitors, and 1 μg of protein was quantified using the Bradford assay [64]. The proteins were separated using 12% SDS-PAGE and moved to PVDF membranes. After blocking with 5% skimmed milk powder in 1X TBS containing 0.1% Tween-20 for 2 h, the PVDF membranes were incubated overnight at 4 °C with rabbit primary antibodies against NLRP3 (1:2000, AdipoGen Life Sciences, San Diego, CA, USA), mature IL-1β (IL-1β p17; 1:1000, Cell Signaling Technology), pro-IL-1β (IL-1β p31; 1:1000, Abcam, Cambridge, UK), cleaved caspase-1 (caspase-1 p20; 1:1000, AdipoGen Life Sciences), pro-caspase-1 (caspase-1 p48; 1:1000, Abcam), and β-actin (1:10,000, Bethyl Laboratories, Montgomery, TX, USA) [63]. The membranes were washed and then reacted with HRP-conjugated secondary antibodies at room temperature (20 ± 5 °C) for 2 h. The membranes were detected using enhanced chemiluminescence, and the target protein bands were observed manually by developing X-ray films or using the LAS-500 mini imager (General Electric, Boston, MA, USA). The expression levels of target proteins were analyzed using ImageJ (1.51j8).

### 4.8. DNCB-Induced AD Mice

A previous study [23] provided an experimental plan to investigate the impact of MAEO on DNCB-induced AD-like mice. In brief, the dorsal skin of the mice was shaved using shaving cream and a clipper, and the mice were divided into three groups of ten mice each: a group that received no treatment (normal), a group that was sensitized with DNCB (control), and a group that was treated with 1% MAEO. To cause AD, DNCB, a compound that causes allergic dermatitis in mice, was diluted to 1% in a mixture of acetone and olive oil (3:1). The dorsal skin and ears of mice were sensitized with 200 μL and 20 μL of 1% DNCB twice a week, resulting in the appearance of AD symptoms, including erythema, edema, and papulation [65]. Seven days after shaving, the dorsal skin and ears were treated daily with either 1% MAEO or olive oil. To maintain AD symptoms, 0.4% DNCB was applied every other day for 2 weeks. The progress of AD symptoms was evaluated every 4 days by measuring the dermatitis score, ear thickness, and transepidermal water loss (TEWL). The assessment of DNCB-induced AD lesions was conducted by applying the SCORing Atopic Dermatitis (SCORAD) index, which scored the severity of erythema, edema, excoriation, and lichenification of the skin on a scale from 0 (no lesion) to 3 (severe) [66]. Ear thickness was measured with a digital micrometer (Mitutoyo Co., Tokyo, Japan) and the TEWL was evaluated using a GPSKIN barrier research solution-II (Gpower Inc., Hanam, Republic of Korea).

### 4.9. Histological Observation

As mentioned in the experimental schedule, the dorsal skin tissue of each mouse was collected on the final day. The skin tissues were preserved in 10% formalin and subsequently embedded in paraffin before being cut into sections [67]. Each section was stained with hematoxylin and eosin (H&E) and toluidine blue (TB), and light microscopy (Olympus, Tokyo, Japan) was used to observe and analyze the histological images. Epidermal thickness was evaluated at a magnification of 200× by examining the H&E-stained sections [68]. The number of mast cells, evaluated by TB staining, was counted in three randomly chosen sections [69].

### 4.10. Double-Immunofluorescence

Paraffin-embedded skin tissue sections were subjected to immunofluorescence staining. Skin sections of 3 µm thickness were deparaffinized and exposed to 0.01 M citrate buffer for antigen retrieval [70]. Immunofluorescence labeling of skin tissue sections was conducted by colabeling with primary antibodies against F4/80 (1:100) and NLRP3 (1:200) or NLRP3 (1:200) and ASC (1:200), followed by incubation with a secondary antibody labeled with AF488 or Cy3 (1:1000, Jackson ImmunoResearch, West Grove, PA, USA) and counterstaining with DAPI (Carl Roth, Karlsruhe, Germany) for 2 h [71]. The immunofluorescently labeled sections were visualized under a confocal microscope (Eclipse A1 Plus, Nikon, Japan) and the average number of positively stained cells was quantified based on three images (200 μm × 200 μm) in a blinded manner [72].

### 4.11. Statistical Analysis

The measurements were performed in triplicate and expressed as the means ± standard error of the mean (S.E.M.). The data analysis was conducted using GraphPad Prism Version 8.0 (GraphPad, La Jolla, CA, USA). Two-way analysis of variance (ANOVA) of one-way ANOVA, followed by a Student—Newman—Keuls test, was used for multiple comparisons, and statistical significance was considered at *p* < 0.05.

## Figures and Tables

**Table 1:** Composition of MAEO.

	Compound Name	Retention Time	Area (%)
1	α-Pinene	8.288	0.43
2	β-Phellandrene	9.731	0.28
3	β-Pinene	9.898	1.12
4	3-Octanol	10.659	0.36
5	Limonene	11.751	3.05
6	Eucalyptol (Cineole)	11.848	0.06
7	β-Ocimene	11.988	0.32
8	trans-β-Ocimene	12.336	0.23
9	Linalool	14.147	0.26
10	Isopulegol	15.772	0.22
11	Menthone	16.113	25.98
12	Neoisomenthol acetate	16.424	2.46
13	Isopulegone	16.585	0.23
14	Menthol	16.897	35.67
15	2,3-Dimethyl-2-cyclopenten-1-one	18.193	0.11
16	Hexen-3-yl Valerate	18.295	0.21
17	Menthone	18.455	0.21
18	Piperitone oxide	18.830	0.44
19	Piperitone	18.964	9.44
20	γ-Diosphenol	19.199	0.02
21	Neoisomenthol acetate	19.922	8.74
22	Diosphenol	20.147	0.10
23	β-Bourbonene	22.404	0.15
24	β-Elemene	22.535	0.05
25	Jasmone	22.674	0.04
26	p-Menthane-1,2,3-triol	23.069	1.93
27	Caryophyllene	23.356	0.89
28	p-Menthane-1,2,3-triol	23.519	0.12
29	Dihydroumbellulone	23.605	0.45
30	Piperitone	24.075	3.85
31	α-Caryophyllene	24.267	0.12
32	Bicyclosesquiphellandrene	24.427	0.31
33	β-Cubebene	24.905	0.84
34	Rose butanoate	24.992	0.03
35	Elixene	25.262	0.05
36	Butylated Hydroxytoluene (BHT)	25.384	0.09
37	γ-Cadinene	25.679	0.04
38	β-Cadinene	25.783	0.07
39	Calamenene	25.860	0.09
40	α-Muuroladiene	26.241	0.05
41	Germacrene-D-4-ol	27.211	0.08
42	Cubenol	28.114	0.11
43	α-Cadinol	29.026	0.08
44	Phytone	32.858	0.04
	Total	99.42	

## Data Availability

Not applicable.

## Abbreviations

AD, atopic dermatitis; Dexa, dexamethasone; DNCB, dinitrochlorobenzene; IL, interleukin; NF-κB, nuclear factor-kappa B; LPS, lipopolysaccharide; ATP, adenosine triphosphate; MAEO, *Mentha arvensis* essential oil; BMDMs, bone marrow-derived macrophages; NLRs, nucleotide-binding oligomerization domain-like receptors; NLRP3, NLR family pyrin domain containing 3; ASC, apoptosis-associated speck-like protein containing a caspase activation and recruitment domain.
